# Are sexually selected traits affected by a poor environment early in life?

**DOI:** 10.1186/s12862-016-0838-2

**Published:** 2016-12-01

**Authors:** Regina Vega-Trejo, Michael D. Jennions, Megan L. Head

**Affiliations:** 1 Division of Ecology and Evolution, Research School of Biology, The Australian National University, Acton, ACT 2601 Australia; 2 Wissenschaftskolleg zu Berlin, Wallotstraße 19, Berlin 14193 Germany

**Keywords:** Age, Catch-up growth, Diet, Mosquitofish, Sperm

## Abstract

**Background:**

Challenging conditions experienced early in life, such as a restricted diet, can detrimentally affect key life-history traits. Individuals can reduce these costs by delaying their sexual maturation, albeit at the price of the later onset of breeding, to eventually reach the same adult size as individuals that grow up in a benevolent environment. Delayed maturation can, however, still lead to other detrimental morphological and physiological changes that become apparent later in adulthood (e.g. shorter lifespan, faster senescence). In general, research focuses on the naturally selected costs of a poor early diet. In mosquitofish (*Gambusia holbrooki*), males with limited food intake early in life delay maturation to reach a similar adult body size to their well-fed counterparts (‘catch-up growth’). Here we tested whether a poor early diet is costly due to the reduced expression of sexually selected male characters, namely genital size and ejaculate traits.

**Results:**

We found that a male’s diet early in life significantly influenced his sperm reserves and sperm replenishment rate. Shortly after maturation males with a restricted early diet had significantly lower sperm reserves and slower replenishment rates than control diet males, but this dietary difference was no longer detectable in older males.

**Conclusions:**

Although delaying maturation to reach the same body size as well fed juveniles can ameliorate some costs of a poor start in life, our findings suggest that costs might still arise because of sexual selection against these males. It should be noted, however, that the observed effects are modest (Hedges’ *g* = 0.20–0.36), and the assumption that lower sperm production translates into a decline in fitness under sperm competition remains unconfirmed.

**Electronic supplementary material:**

The online version of this article (doi:10.1186/s12862-016-0838-2) contains supplementary material, which is available to authorized users.

## Background

Conditions experienced early in life affect life-history trajectories [1, 2]. In particular, lower growth rates due to limited food availability during development tend to reduce adult body size [3]. In some species, however, individuals reduce the potential fitness costs of smaller adult body size. For example, if food again becomes available, they compensate by accelerating their growth (compensatory growth). Alternatively, they delay maturity to attain the same adult size as well-fed individuals (catch-up growth; reviews: [4, 5]). There are usually clear benefits to large adult size, such as increased survival and higher reproductive success (e.g. [6]), but reaching the same size as better fed individuals might generate other costs (e.g. [7]). An obvious cost of catch-up growth is a longer generation time and, if there is seasonal breeding, a shorter reproductive lifespan [8, 9]. More subtle costs arise when elevated or extended growth produces developmental abnormalities that can, for example, increase the risk of predation, decrease immune function, and lower resistance to stressors (e.g. [4, 10–12]). Poor nutrition early in life has been shown to adversely affect adult behaviour [11, 13], locomotor performance [14], functional morphology [15, 16], and key adult life-history traits [17–20]. But even if there are no obvious effects, a poor start in life can still decrease fitness. For example, individuals reared on a restricted diet might be morphologically indistinguishable from those reared on a standard diet, but have shorter telomeres or lower plasma antioxidant levels [21–23], which should reduce their adult lifespan (but see [2]).

A major life history allocation decision is how to invest in naturally and sexually selected traits. In general, however, we know little about how conditions early in life affect allocation of resources to adult life history traits ([24], but see: [25]). In particular, far more studies have investigated the effects of early diet on naturally rather than sexually selected traits, (but see: [26–29]). This is surprising because variation in male lifetime reproductive success is often predominantly attributed to differences in mating success (i.e. sexual selection; [30]). Most sexually selected traits are under strong directional selection, and food availability is often a major determinant of their condition-dependent expression. Pre-copulatory sexually selected traits (i.e. those that determine mating success) can be detrimentally affected by poor early nutrition. For example, male eye-span in stalked-eyed flies and song repertoire size in great reed warblers, which are both traits that affect female mate choice, are negatively affected by nutritional stress during development [31, 32]. This reduced investment could reflect a life history trade-off between sexually and naturally selected traits. For example, when early nutrition is poor, males sometimes reduce investment in sexual ornaments to maintain their oxidant defence systems [21, 29]. Similar trade-offs could also affect investment into different sexually selected traits [33, 34]. Male reproductive success usually depends on both mating success and sperm competitiveness (e.g. [35–37]). Given lower resource availability, males might invest differently in traits under pre-copulatory and post-copulatory sexual selection (e.g. [38, 39]). This could shift the relative allocation of resources to sperm competitiveness versus attractive ornaments [40]. For example, greater investment in larger body size or weaponry can result in smaller testes and ejaculates [41, 42].

The condition-dependence of sperm traits has been examined in several species, but this is usually due to short-term effects of manipulating the adult diet (e.g. [43–45]). Fewer studies, especially of vertebrates, have tested how a restricted juvenile diet affects sperm traits (but see: [35, 40, 46]). Male fertilization success is highly dependent on resource allocation to traits that are under post-copulatory sexual selection, especially when sperm competition is intense [47]. In such species, males tend to have relatively larger testes that produce more sperm [48, 49]. Of course, sperm production is not the sole predictor of sperm competitiveness. It can also depend on sperm viability, swimming speed, and even sperm length [50, 51]. Since ejaculates are costly to produce [44, 52], it follows that a poor juvenile diet could negatively affect the number, quality, and rate of sperm production (e.g. [53, 54]).

Here we test whether the juvenile diet of male mosquitofish (*Gambusia holbrooki*), despite having no effect on adult body size, affects two sexually selected adult traits: ejaculate production and genital size. In two earlier studies we showed that males with limited food intake as juveniles reach a similar size to males on a normal diet due to delayed maturation [55, 56]. Additionally, we showed that males with a poor start in life are less attractive to females than those reared on a regular diet [57]. Mosquitofish are poecillid fishes characterised by frequent, coercive mating attempts and intense sexual selection, including sperm competition [54, 58, 59]. Males internally inseminate females using a modified anal fin as an intromittent organ, the ‘gonopodium’ [60]. Several recent studies have linked greater gonopodium length to increased male reproductive success in *G. holbrooki* ([61, 62] but see [63]). We ask whether males initially raised on a restricted diet incur sexually selected costs, despite catch up growth, due to the production of lower quality ejaculates, or development of a shorter gonopodium.

## Methods

Fish were bred as part of a larger study to test how inbreeding and food restriction affect compensatory growth [55]. We found no effects of inbreeding on any of the measured life history variables (growth trajectories and adult size). Here, we are specifically interested in whether early diet restriction influences sexually selected traits so, for clarity, we analyse the data excluding inbreeding from our models. Including inbreeding does not qualitatively alter our results because it had very small, non-significant effects. These are discussed elsewhere (J. Marsh, R. Vega-Trejo, M.L. Head, and M.D. Jennions ‘in preparation’).

We used mosquitofish descended from females captured in ponds in Canberra, Australia (35°14’27”S, 149°5’27”E and 35°14’13”S, 149° 5’55”E) in February-March 2013. Full methods are described in Vega-Trejo et al. [64]. In brief, in each experimental block we mated individuals from two families (e.g. A and B in block 1, C and D in block 2 and so on). Brothers and sisters from full sibling families were paired to create inbred (AA, BB) and outbred offspring with reciprocal male–female crosses (AB, BA; i.e. four cross-types). We set up 29 blocks and with one male and one to four full sisters per cross type. The resultant offspring were reared individually in 1 L tanks until maturity (*N* = 453 males) under a 14:10 h photoperiod at 28 °C. Males underwent a diet manipulation for 21 days between days 7 and 28 post-birth. Fish on the control diet were fed *ad libitum* with *Artemia* nauplii twice daily (i.e. standard laboratory feeding regime) whereas fish on the restricted diet were fed 3 mg of wet mass *Artemia* nauplii once every other day. We have previously shown that this restricted diet leads to minimal growth without elevating juvenile mortality [56]. Broods were split evenly between the control and restricted diet treatments. Males were considered mature when their gonopodium was translucent with a spine visible at the tip [65, 66]. These changes are associated with spermatogenesis in the testes [60, 66]. We have previously shown that inbreeding (i.e. mating with full-sibs) reduces the number of offspring at birth, but with no detectable effect on their likelihood of breeding, gestation time, offspring size at birth or growth rate [64]. In addition, there is no difference in juvenile survival between males on the control and the restricted diet (i.e. GLMM ran for food treatment: *P* = 0.952; [55]). We collected body size and sperm data from mature males (range: 2–18 weeks post-maturity). We define ‘developmental time’ as the number of days that males took to reach maturity, and ‘adult age’ as the post-maturation age at which sperm was extracted (i.e. total age – developmental time). Developmental time was 78.6 ± 34.3 days for males in the control treatment and 99.4 ± 37.5 for males in the restricted diet treatment. Adult age was 81.0 ± 17.1 days for males on the control diet, and 70.4 ± 21.4 days for males on the restricted diet (mean ± SD).

### Sperm traits

We tested 453 males from 192 broods. Sperm was collected on three occasions: on day 0 we stripped virgin males of sperm (see below) to measure their maximum sperm reserves; one day later we stripped males to measure their sperm replenishment rate (i.e. sperm production over 24 h); on day 3 we stripped males to measure sperm velocity. We also calculated the proportion of sperm replenished (= number of sperm at day 1/number of sperm at day 0), which we arcsine-transformed to normalize the error distribution.

### Sperm collection

To strip ejaculates, males were anaesthetized in ice-cold water. The male was then placed on a glass slide (coated with 1% polyvinyl alcohol solution (PVA) to prevent sperm bundles sticking to the slide) under a dissecting microscope. His gonopodium was swung forward and we applied gentle pressure to the abdomen to eject all the available sperm. We transferred the ejaculate to an Eppendorf tube with 100–900 μL of extender medium (207 mM NaCl, 5.4 mM KCl, 1.3 mM CaCl_2_, 0.49 mM MgCl_2_, 0.41 mM MgSO_4_, 10 mM Tris, pH 7.5). The amount of medium varied depending on the amount of ejaculate stripped to obtain accurate sperm counts, which require an intermediate sperm concentration. Sperm remain quiescent in this solution until activated [67]. Sperm counts and velocity measures were taken within 30 min of sperm collection (see [51] for further details). After the procedure each male was returned to his individual tank. Sperm collection was done blind to diet treatment by RVT.

### Sperm number

To estimate the number of sperm we vortexed the sperm solution for 1 min and then mixed it repeatedly with a pipette (20–30 times) to break up sperm bundles and distribute the sperm evenly throughout the sample. We placed 3 μl of solution on a 20 micron capillary slide (Leja) and counted the sperm using a CEROS Sperm Tracker (Hamilton Thorne Research, Beverly, MA, USA) under 100× magnification. We counted five subsamples per sample. We estimated repeatability following Nakagawa and Schielzeth [68] using the *rptR* package in *R 3.0.2* [69]. Repeatability was very high for sperm number (sperm at day 0: *r* = 0.85 ± 0.01 SE; sperm at day 1: *r* = 0.91 ± 0.006 SE). The mean of the five subsamples was used for further analyses. The threshold values defining cell detection were predetermined as elongation percentage 15–65, head size 5–15 μm, and the static tail filter was set off. Sperm were counted blind to male treatment.

### Sperm velocity

For each ejaculate we analysed three samples. For each sample we collected 3 μL of the diluted sperm (above) and placed this in the centre of a cell of a 12-cell multitest slide (MP Biomedicals, Aurora, OH, USA) previously coated with 1% PVA. The sample was then activated with a 3 μL solution of 150 mM KCl and 2 mg ml^−1^ bovine serum albumin [70] and covered with a cover slip. We analysed sperm velocity within 30 s of activation for three subsamples to increase the number of velocity measures. We used an average of 109.3 ± 3.5 SE sperm tracks per ejaculate (minimum 10 sperm tracks/male). We excluded six of 399 available males from the velocity analysis because they had fewer than 10 sperm tracks. We recorded two standard measures of sperm velocity: (1) average path velocity (VAP): the average velocity over a smoothed cell path and (2) curvilinear velocity (VCL): the actual velocity along the trajectory using a CEROS Sperm Tracker. The threshold values defining static cells were predetermined at 20 μm/s for VAP and 15 μm/s for VCL. Repeatability was high for both parameters (VAP: *r* = 0.65 ± 0.02 SE; VCL: *r* = 0.58 ± 0.03) and we used the mean of the three subsamples in our analyses. Due to the near perfect correlation between VAP and VCL (*r* = 0.961, *P* < 0.001), as found in most comparable studies (e.g. [71–73]), we only use VAP in our analyses.

### Male morphology

All males were measured a week after sperm extraction. Males were anaesthetized by submersion in ice-cold water for a few seconds to reduce movement and then placed on polystyrene with a microscopic ruler (0.1 mm gradation) and photographed. We measured male standard length (SL = snout tip to base of caudal fin) and gonopodium length using *Image J* [74].

### Statistical analysis

We removed one of 453 males from the analysis because he had a higher number of sperm on day 1 than day 0 indicating that not all sperm were collected during the first extraction. To analyse the effect of diet treatment on male sexual traits we used generalized linear mixed models (GLMM). We constructed separate models for each of our five response variables: gonopodium length, number of sperm at day 0, number of sperm at day 1 (i.e. replenishment rate), proportion of sperm replenished (arc-sine transformed), and VAP. In each model we included diet as a fixed factor, and male standard length, development time, and adult age as fixed covariates, as well as all two-way interactions with diet. Gonopodium length and body size were log-transformed. Adult age was not included in the model for gonopodium length as there is little post-maturity growth in *G. holbrooki*.

There were significant bivariate correlations between development time and body size (*r* = 0.62, *P* < 0.001) as well as development time and adult age (*r* = −0.77, *P* < 0.001). The former reflects a biological relationship and the latter is due to a logistic constraint (i.e. having to terminate the experiment). Even so, these correlations were not so large as to preclude including all three terms as covariates in a GLMM due to colinearity problems: running each model with one covariate at a time produced comparable effect sizes for focal terms.

More importantly, we needed to take into account that the mean development time differed significantly between the diets because of catch up growth by males on the restricted diet (GLMM with diet as the single fixed factor, and random factors as below: *P* <0.001). Including development time as a raw covariate could obscure a main effect of diet (i.e. it is a covariate measured post-treatment *sensu* A Gelman and J Hill [75], p.188 that might causally mediate any diet effect because it varies due to the diet itself). We therefore standardised developmental time *within* each diet treatment (both treatments: mean = 0, S.D =1). We also standardised male standard length *within* each diet for ease of interpretation of the results. However, male size did not differ between the diet treatments (*P* = 0.451; see Results) so the use of unstandardised male size yields almost identical results. In contrast, adult age was not standardised *within* diet treatments because it varied depending on when we were able to test males. Instead we standardised adult age *across* the study to aid in interpretation (i.e. the intercept is the value for an average aged male; [76]). Although mean adult age at testing differed significantly between the two diets, there was a large overlap in values (Additional file 1).

Centring the covariates within each diet affects their interpretation. The effect of development time (or its interaction with diet) should be interpreted relative to that of other males on the same diet. The main effects of diet are interpretable as those for a male of average size and development time for its treatment type, but of average age for males across the entire study (see [76]).

In all the GLMMs we specified a Gaussian error distribution and checked the distribution of model residuals to ensure this was appropriate. The use of Poisson error (for count data) and binomial error (for proportions) provided a worse fit to the data than the use of Gaussian error on raw or transformed dependent variables. Each model was fitted using the *lme4* package in *R 3.0.2* software with block, maternal identity, and sire identity as random factors (see [64]). All model terms were tested for significance using the Anova function in the *car* package specifying Type III Wald chi-square tests. Model simplification (i.e. removing non-significant interactions and main terms) did not change our results. Marginal R^2^ refers to the variance explained by fixed factors in a model, estimated on the link scale [77]. We present the marginal R^2^ (ΔR^2^) to show the decline when each fixed effect was dropped from the full model.

We also calculated the effect size (Hedges’ *g*) as the standardized difference between males on the control and restricted diets for the measured traits following Rosenberg et al. [78].

Figures are presented using raw data but with model estimates for regression lines, unless otherwise stated. Summary statistics are presented as mean ± SE.

## Results

The correlations between the four ejaculate traits are provided in Table 1. Diet treatment means for the five male traits and effect sizes for diet are provided in Table 2. Parameter estimates from the GLMM models are provided in Table 3.

**Table 1:** Correlations between sperm traits measured

	Number of sperm at day 1	Proportion of sperm replenished	Sperm velocity (VAP, μm/s)
Number of sperm at day 0	0.468 (<0.001)	−0.117 (0.015)	0.055 (0.271)
Number of sperm at day 1		0.664 (<0.001)	0.032 (0.526)
Proportion of sperm replenished			0.059 (0.246)

Estimates are followed by *p*-values in brackets. *N* = 452 males or 393 males (for sperm velocity)

**Table 2:** Treatment means ± SE (N) for the five traits measured

	Control diet	Restricted diet	Hedges’ *g*
Gonopodium length (mm)	6.94 ± 0.05 (223)	7.03 ± 0.04 (226)	0.137
Number of sperm at day 0 (×10^5^)	194.58 ± 6.37 (225)	176.20 ± 6.14 (227)	0.196
Number of sperm at day 1 (×10^5^)	62.36 ± 2.72 (225)	47.92 ± 2.67 (227)	0.356
Proportion of sperm replenished	0.35 ± 0.02 (225)	0.29 ± 0.01 (227)	0.268
Sperm velocity (VAP, μm/s)	83.10 ± 1.11 (207)	81.88 ± 1.12 (186)	0.073

**Table 3:** Results from mixed models with parameter estimates and chi square (*χ* ^2^) tests for food treatment, size, developmental time, and adult age

	Predictor	Estimate	SE	*χ* ^2^	*P*	∆R^2^
Gonopodium length [ln (mm)]						
Control diet (N = 223)	Intercept	0.838	0.002	152650	<0.001	
Restricted diet (N = 226)	Diet (restricted)	0.008	0.002	12.670	**<0.001**	0.047
	Size	0.041	0.002	410.140	**<0.001**	0.332
	Developmental time	0.003	0.002	2.553	0.110	0.032
	Diet × Size	−0.020	0.003	43.952	**<0.001**	0.032
	Diet × Developmental time	0.005	0.003	3.193	0.074	0.002
Number of sperm at day 0 (total count ×10^5^)						
	Intercept	197.389	8.502	538.991	<0.001	
Control diet (N = 225)	Diet (restricted)	−10.103	8.904	1.2875	0.257	0.036
Restricted diet (N = 227)	Size	25.761	6.978	13.627	**<0.001**	0.040
	Adult age	−19.425	10.606	3.3546	0.067	0.033
	Developmental time	−26.608	10.768	6.1057	**0.013**	0.009
	Diet × Size	0.517	11.465	0.002	0.964	<0.001
	Diet × Adult age	53.202	13.371	15.831	**<0.001**	0.028
	Diet × Developmental time	22.339	14.83	2.269	0.132	0.004
Number of sperm at day 1 (total count ×10^5^)						
	Intercept	62.4092	3.1771	385.8565	<0.001	
Control diet (N = 225)	Diet (restricted)	−10.9804	3.8738	8.0347	**0.005**	0.023
Restricted diet (N = 227)	Size	12.9494	3.0323	18.237	**<0.001**	0.057
	Adult age	−5.3597	4.568	1.3767	0.241	0.012
	Developmental time	−21.5316	4.8045	20.0842	**<0.001**	0.045
	Diet × Size	0.8841	5.3062	0.0278	0.868	<0.001
	Diet × Adult age	14.6051	5.8465	6.2404	**0.012**	0.01
	Diet × Developmental time	9.4495	6.6831	1.9992	0.157	0.003
Proportion of sperm replenished						
Control diet (N = 225)	Intercept	219.386	9.188	570.1931	<0.001	
Restricted diet (N = 227)	Diet (restricted)	−30.524	12.415	6.0454	**0.014**	0.013
	Size	22.047	9.611	5.2624	**0.022**	0.02
	Adult age	−4.278	14.272	0.0898	0.764	<0.001
	Developmental time	−55.801	15.094	13.6668	**<0.001**	0.047
	Diet × Size	6.203	16.712	0.1378	0.711	<0.001
	Diet × Adult age	8.97	18.707	0.23	0.632	<0.001
	Diet × Developmental time	9.292	21.251	0.1912	0.662	<0.001
Sperm velocity (VAP, μm/s)						
Control diet (N = 207)	Intercept	85.578	1.261	4603.423	<0.001	
Restricted diet (N = 186)	Diet (restricted)	−4.569	1.652	7.650	**0.006**	0.034
	Size	−0.308	1.312	0.055	0.814	<0.001
	Adult age	−8.066	1.932	17.426	**<0.001**	0.062
	Developmental time	−0.293	1.999	0.022	0.883	0.007
	Diet × Size	1.552	2.334	0.442	0.506	<0.001
	Diet × Adult age	2.552	2.529	1.018	0.313	0.003
	Diet × Developmental time	−3.568	2.909	1.505	0.220	0.003

*P*-values in bold indicate significant values. Covariates were standardised within food treatment. The sample sizes for control and restricted diets are given for each response variable. ∆R^2^ shows the change in marginal R^2^ when each fixed effect is dropped from the model

### Effect of treatment on male morphology

**Fig. 1:**
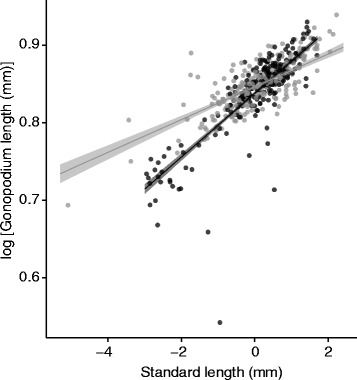
Effect of diet treatment on the relationship between male body size and gonopodium length. Standard length is standardised within each diet. *Black* symbols and lines represent the control diet, *grey* symbols and lines represent the restricted diet. *Lines* represent simple model predictions. *Grey* shading represents 95% confidence intervals

There was no significant difference between the diet treatments in male body size at maturity (control: 23.52 ± 0.14 mm; restricted diet: 23.35 ± 0.11 mm; *t* = 0.92, *P* = 0.36). Against expectations (see [56]), males on the restricted diet had a significantly *longer* gonopodium than those on the control diet if they were of average or smaller body size, but a shorter gonopodium if they were of above average size (diet × size: *P* < 0.001; Table 3; Fig. 1). Correcting for body size, males that took relatively longer to mature on a given diet did not have a significantly longer gonopodium (*P* = 0.110), irrespective of the diet type (diet × development time: *P* = 0.074).

### What influences sperm traits?

#### Initial sperm reserves (Day 0)

**Fig. 2:**
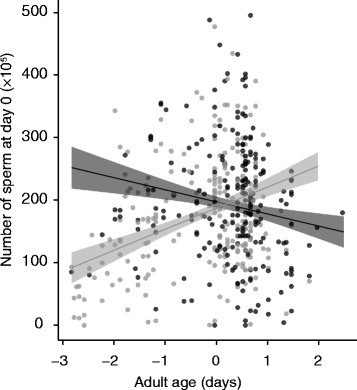
Effect of diet treatment on the relationship between number of sperm at day 0 and adult age. Number of sperm represent total counts, adult age is standardised across diet. *Black* symbols and *lines* represent the control diet, *grey* symbols and *lines* represent the restricted diet. *Lines* represent simple model predictions. *Grey* shading represents 95% confidence intervals

As expected, larger males had significantly greater initial sperm reserves (*P* < 0.001), irrespective of their diet (diet × size interaction: *P* = 0.964). However, diet significantly influenced how sperm reserves changed with age (diet x adult age: *P* < 0.001). Initial sperm reserves of males on the control diet tended to decrease with age (*P* = 0.067), while there was a significant increase with age for males on the restricted diet (Estimate ± SE for males on the restricted diet: 33.777 ± 9.306, *P* <0.001; Fig. 2).

Although the two slopes intersect, interpretation of the age-dependent change in sperm reserves is best confined to stating that, when younger, males on the restricted diet have lower sperm reserves than those on the control diet. This is because there are relatively few data points for males on the restricted diet when standardised male age exceeds 1 (see Fig. 2). There was also a significant effect of development time on initial sperm reserves (*P* = 0.013), irrespective of diet (diet × development time: *P* = 0.132).

#### Sperm replenishment rate (Day 1)

Larger males had significantly higher sperm replenishment rates than smaller males (*P* < 0.001), irrespective of their diet (diet × size: *P* = 0.868); but, controlling for body size, males that took longer to develop had a significantly lower sperm replenishment rate (*P* < 0.001) irrespective of their diet (diet × development time: *P* = 0.157). A male of average size and development time for its diet, and average age for the study, that was reared on the control rather than the restricted diet had a significantly higher replenishment rate (*P* = 0.005). There was, however, also a significant difference between the diets in how age related to replenishment rate (diet x adult age: *P* = 0.012): males on the control diet had no significant change in replenishment rate with age (*P* = 0.241; Table 3), while replenishment rate increased significantly with age for males on the restricted diet (Estimate ± SE for males on the restricted diet: 9.245 ± 3.977, *P* = 0.020).

Overall, males on the control diet replenished a significantly greater proportion of their initial sperm reserves within 24 h than those on the restricted diet (*P* = 0.014). The proportion replenished was significantly greater for larger males (*P* = 0.022), and for males with a shorter development time for their diet type (*P* < 0.001), but there was no significant effect of male age (*P* = 0.764). All these relationships held irrespective of diet (interactions with diet: all *P* > 0.632).

**Fig. 3:**
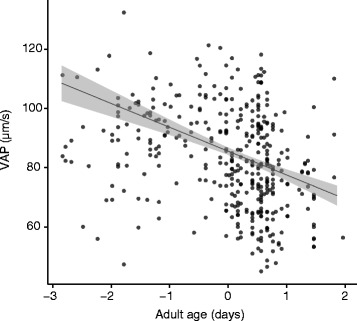
The relationship between VAP (average path velocity) and adult age. VAP is given in μm/s, adult age is standardised across diet. The *line* represents model predictions. *Grey* shading represents 95% confidence intervals

Finally, males on the control diet had significantly faster swimming sperm than did males on the restricted diet (*P* = 0.006). Sperm velocity also decreased significantly with adult age (*P* < 0.001; Fig. 3), but was unrelated to development time or body size. All these relationships held irrespective of diet (interactions with diet: all *P* > 0.220).

## Discussion

Nutritional constraints early in life can lower an individual’s fitness due to changes in their adult performance. Like many species [5, 24], juvenile mosquitofish that experience food restrictions early in life extend their development time to attain a similar body size to individuals on a regular diet (see [55, 56]). But do additional costs arise despite this equivalence in adult body size? Here we tested whether a poor juvenile diet has sexually selected costs for male *Gambusia holbrooki* due to a decline in ejaculate quality and the development of shorter genitalia. We found that early diet had a significant influence on initial sperm reserves, sperm replenishment rate, the proportion of sperm replenished, and on sperm velocity. Shortly after maturation males that had a restricted diet during development had smaller sperm reserves and lower sperm replenishment rates early in adulthood than males that did not have a restricted diet. However, these effects were not detectable for older males (see Fig. 2). In contrast, and unexpectedly, males on the control diet had relatively shorter gonopodia than those on the restricted diet, when they were of small or average body size. Our results, combined with those from our previous studies [56, 57], suggest that a poor diet early in life not only has the immediate cost of delayed maturation, but might impose additional costs if lower sperm production, slower swimming sperm, and deviations from the normal gonopodium-body size allometry reduce male fertilisation success under sperm competition.

Sperm production is condition dependent in a variety of species. When the diet of adult males is restricted they tend to have smaller sperm reserves (e.g. [44, 73, 79–81]) and lower sperm replenishment rates (e.g. [54]). There are, however, far fewer studies that explore the effects of a poor juvenile diet on sperm reserves and sperm replenishment rates (but see: [35, 40]). We found that both sperm reserves and replenishment rates were affected by a male’s early diet in an age-dependent manner. Thus, in addition to the immediate condition-dependence of sperm production reported in previous studies, we have shown that early diet restriction can have much longer-term effects on sperm production. Whether the relatively small (albeit significant) effects that diet has on sperm production translate into differences in reproductive success remains to be tested. The effect size for the direct effect of diet on sperm production and reserves ranges from *g* = 0.20 to 0.36. To put this in context, by convention, effects of a 0.1 and 0.3 standard deviation change in means are usually described as ‘small’ and’medium’ respectively [82].

Although many factors affect ejaculate competitiveness under sperm competition, sperm number still tends to predict variation in male fertilization success under sperm competition [83–85]. For example, it is a good predictor of fertilization success in another poeciliid fish, the guppy [51]. More generally, a consistent pattern in comparative analyses of diverse taxa is that species with more intense sperm competition have larger testes, and produce more sperm [86–88]. This strongly suggests that sperm production is sexually selected under sperm competition. Our results imply that males that experience a restricted juvenile diet, even if they mate as often as males that had a regular diet, will have lower lifetime reproductive success due to reduced sperm competitiveness. Although sperm reserves increase with time since sexual maturity (here and [89]), males experiencing restricted diets during development may still be disadvantaged because they take longer to reach full sperm production. Additionally, it is worth noting that at our study site adult mosquitofish males do not overwinter [90]. The limited time available to breed (November-March) should favour males that reach full sperm reserves at maturation. However, although we believe that these arguments are compelling, to confirm that the lower sperm production we have reported affects male fitness we still need direct tests of fertilization success whereby males reared on a restricted and normal juvenile diets compete. It would also be interesting to look at how early diet influences the composition of seminal fluids, as this may influence sperm competiveness, for instance by altering sperm longevity ([91], but see [92]) and thus the potential for sperm to be stored over winter.

Our findings for juvenile dietary effects on ejaculatory traits are analogous to those in studies showing that variation in early nutrition due to parental care affects other adult sexual traits that determine male reproductive success [93–95]. For example, in a dung beetle, developing larvae depend on nutrients provided by their parents which affects male body and horn size and thereby their mating success [93]. In some cases the diet or conditions that parents’ experience is transmitted to their own offspring (i.e., transgenerational effects: [1, 96, 97]), and can thereby affect their mating success. For example, in birds the amount of carotenoids available to mothers influences what they can deposit into egg yolks, which can then affect their sons’ adult ornamental coloration [98]. Our results highlight that, regardless of whether variation in the early nutritional environment is determined by parental care, an individual’s resource acquisition ability, or the habitat into which it is born, the effects on offspring fitness are potentially far reaching, and could extend into adulthood. More specifically, we suggest that it could be worthwhile to test for parental effects on sperm production.

Intriguingly, sperm velocity decreases with adult age in *G. holbrooki*. Sperm quality is expected to decline with age due to lower fertilising efficiency and/or the genetic quality of sperm produced by ageing males [99]. This expectation is supported by studies showing that sperm velocity deteriorates with male age (e.g. [100–102]). The lower sperm quality of older males has been shown to reduce fertilization success under sperm competition in some cases (i.e. when competing with sperm from younger males; e.g. [103]) but not others [92, 104]. There are two possible reasons why older males might have lower quality sperm. One is that old males *produce* lower quality sperm (an effect of male age per se; e.g. [100, 105]). The other is that the sperm of older males deteriorates because it has spent more time in storage (an effect of sperm age; e.g. [106, 107]). In our study all sperm velocity measures were obtained from sperm that were at most three days old, so the observed lower sperm velocity is most likely due to an effect of male rather than sperm age. It is intriguing that sperm numbers increased with age (at least for males on the restricted diet), while sperm velocity declined with age for all males. This suggests that sperm number might be a more important determinant of fertilization success than sperm velocity, and is therefore more likely to be maintained given limited resources. This is supported by data from other poeciliids showing that sperm number is more important than sperm velocity under post-copulatory sexual selection (an effect of sperm age; e.g. [106, 107]). Additionally, unlike studies in other poecillids (see [43]), sperm velocity was significantly negatively affected by a restricted diet, albeit that the effect size was small (Hedges’ *g* = 0.07). This finding is nonetheless interesting given that we only manipulated diet early in life. Again, however, it remains to be directly shown that the observed dietary difference in sperm velocity affects male fertilization success under sperm competition.

Male fitness depends on the ability to acquire mates and gain paternity when females mate multiply (see [39, 43]). Although the quantity and the quality of sperm tends to strongly influence male fertilization success in most taxa, other traits can be important (e.g. genital morphology in dung beetles; [108]). We measured gonopodium length, which affects female mate choice in some poeciliids and has been implicated as a potentially important trait affecting sperm transfer [109–112]. Unexpectedly, we found that small and medium-sized males on a restricted diet early in life had a longer gonopodium, corrected for body size, than those on a regular diet [60, 113]. The fitness consequences of this change in allometry are unclear. A female preference for males with a relatively longer gonopodium has been shown in *G. holbrooki*, but only for large bodied males (see [56] for a different finding). In addition, [109] failed to detect a female preference when using lines of males artificially selected for a relatively larger or shorter gonopodium. Insemination success seems to depend on both male body size and gonopodium length. Males with a relatively longer gonopodium are likely to be more successful, but only when they are large bodied [114]. Paternity studies of males free to compete for females have, however, produced contradictory results. Two studies [61, 62] found that males with a relatively longer gonopodium gained a greater share of paternity, while another study [63] found no difference in the reproductive success of males from lines selected for a relatively longer or shorter gonopodium. Consequently, the effects on male reproductive success of the observed diet-dependent change in relative gonopodium length remain unclear.

## Conclusions

In sum, some ejaculate traits in *G. holbrooki* depend on an interaction between a male’s juvenile diet and his adult age. In a previous study we also showed that early life diet influences male attractiveness in *G. holbrooki* [57]. Together these studies suggest that early diet could have fitness consequences that only become apparent in adulthood. Our findings are similar to those in other species where males on different diets superficially look the same, but differ in social dominance [57], telomere length or plasma antioxidant levels (e.g. [13]). As with these studies it is assumed that the traits affected by diet influence male fitness. However, the actual effects of a poor early diet on adult male reproductive performance remain to be directly tested. Ideally, future studies should directly measure the relative reproductive success of males that undergo a poor start in life in a competitive mating context (but see [62]).

## Additional file

Additional file 1: Shows the overlap in values of adult age between the two diets. (DOCX 49 kb)
