# Supplementary material for: Are sexually selected traits affected by a poor environment early in life?
Source: BMC Evol Biol. 2016 Dec 1;16:263. doi: 10.1186/s12862-016-0838-2 (PMC5134236; doi:10.1186/s12862-016-0838-2)
Supplement: Additional file 1: — Shows the overlap in values of adult age between the two diets. (DOCX 49 kb) [file 12862_2016_838_MOESM1_ESM.docx]

Additional file 1

Histogram of adult age (days since maturation) for control diet (C) and low food diet (L) males.
